# “*PrEP protects us*”: Behavioural, normative, and control beliefs influencing pre-exposure prophylaxis uptake among pregnant and breastfeeding women in Zambia

**DOI:** 10.3389/frph.2023.1084657

**Published:** 2023-04-19

**Authors:** Twaambo Euphemia Hamoonga, Wilbroad Mutale, Lauren M. Hill, Jude Igumbor, Benjamin H. Chi

**Affiliations:** ^1^ School of Public Health, University of Zambia, Lusaka, Zambia; ^2^ School of Public Health, Faculty of Health Sciences, University of the Witwatersrand, Johannesburg, South Africa; ^3^ Gillings School of Global Public Health, University of North Carolina at Chapel Hill, Chapel Hill, NC, United States; ^4^ School of Medicine, University of North Carolina at Chapel Hill, Chapel Hill, NC, United States

**Keywords:** PrEP, beliefs, pregnant, breastfeeding, theory of planned behaviour, intention, Zambia, sub-Saharan Africa

## Abstract

**Background:**

Although pre-exposure prophylaxis (PrEP) is recommended for pregnant and breastfeeding women at elevated HIV risk, uptake has been low in Zambia.

**Methods:**

In in-depth interviews, we explored beliefs about PrEP among 24 HIV-negative pregnant and breastfeeding Zambian women. Thematic analysis was used to identify behavioural, normative and control beliefs likely to influence PrEP uptake.

**Results:**

Most women viewed PrEP as a good method of protecting themselves and their babies from HIV infection. Partners were cited as key referents in decision making about PrEP use. Many women felt that PrEP use was not entirely in their control. Most reported that they would not use PrEP if their partners did not approve. Health care providers with negative attitudes, long distance to clinics, and extended waiting times were cited as barriers to PrEP uptake.

**Conclusion:**

HIV-negative pregnant and breastfeeding women had a positive attitude towards PrEP but barriers to uptake are multifaceted.

## Introduction

In sub-Saharan Africa, pregnancy and breastfeeding are periods of increased risk for HIV acquisition. In two meta-analyses, HIV incidence during these periods were at or above the World Health Organization's threshold for high risk (3.0 infections per 100 person-years) (1, 2). The risk of HIV acquisition during pregnancy has been attributed in part to health facility-related factors (e.g., inadequate education about HIV prevention among antenatal care (ANC) attendees (3, 4)), social and behavioral factors [e.g., low condom utilization (5–8)] and low rates of HIV status disclosure to sexual partners (9, 10). Biological, physiological and immunologic alterations also contribute to the elevated HIV risk observed in pregnant and postpartum populations (11–16).

Biomedical interventions such as pre-exposure prophylaxis (PrEP) have the potential to reduce the risk of maternal HIV acquisition (17) and may play an important role in the elimination of HIV mother-to-child-transmission. The World Health Organization (WHO) endorses the use of PrEP during pregnancy and breastfeeding for HIV-negative women who are at higher risk of HIV acquisition, depending on individual behaviour and the characteristics of sexual partners (17). This recommendation is based on PrEP's efficacy and its safety track record across numerous studies in pregnancy (18). While many national programs have introduced PrEP for pregnant and breastfeeding populations (19), there is need to identify and engage people at risk for HIV and further improve demand for PrEP (20, 21).

The Zambia Ministry of Health first introduced PrEP as a key strategy for HIV prevention in 2016 (22). In line with WHO recommendations, the country's 2020 HIV treatment and prevention guidelines extended the provision of PrEP to HIV-negative pregnant and breastfeeding women at substantial risk for HIV acquisition (23). However, to date, uptake of these services in antenatal and postnatal populations has been limited, with some studies reporting rates as low as 1% (24), despite the high rate of mother-to-child transmission (25). The increasing burden of new infant HIV infections can be traced to incident maternal HIV during pregnancy and breastfeeding (26) which may go undiagnosed and therefore untreated. Taking PrEP during pregnancy and breastfeeding has the potential to reduce the risk of maternal seroconversion and onward mother-to-child transmission of HIV. However, factors that may influence uptake of PrEP in this population largely remain unknown.

To better understand facilitators and barriers to PrEP uptake in antenatal settings, we conducted a qualitative study guided by the Theory of Planned Behaviour (TPB) framework. We applied the TPB to understand behavioural, normative and control beliefs that pregnant and breastfeeding women have about PrEP. The TPB assumes that individuals act rationally, according to their attitudes, subjective norms, and perceived behavioral control. According to this theory, in order to predict whether a person intends to engage in a health behaviour, it is important to know whether the person is in favour of doing it (attitude), how much the person feels social pressure to do it (subjective norm), and whether the person feels in control of the behaviour in question (perceived behavioural control) (27). Strategies that are able to modify these three factors can raise a woman's intention to take PrEP and, by doing so, increase the likelihood that she actually takes up the intervention (28).

## Materials and methods

### Study design and population

In this qualitative study, we recruited HIV-negative pregnant and breastfeeding women from Chipata Level 1 Hospital in Lusaka, Zambia. This government health facility has a catchment population of over 100,000 and an antenatal clinic that attends to about 400–450 new ANC attendees each month. The HIV prevalence among pregnant women attended to at this health facility is approximately 16%, similar to the national prevalence for women (29). Purposive sampling was used to recruit study participants from the Maternal and Child Health clinic, where they were receiving either antenatal or postnatal care services. Pregnant and breastfeeding women aged 18 years or older and with a documented HIV-negative result in their antenatal record, were eligible. We enrolled 24 participants between November 2020 and March 2021. The sample size was determined using the principle of theoretical saturation, the point where additional interviews did not add any new insights on beliefs that women held about PrEP (30).

### Data collection

We used a semi-structured questionnaire which had two components: the first part which was structured was used to collect data on socio-demographic characteristics of study participants (i.e., age, educational attainment, employment status and marital status), risky sexual behaviour as well as knowledge about PrEP. The second part which took the form of an interview guide was developed based on the Theory of Planned Behaviour questionnaire (27). The Theory of Planned Behaviour questionnaire has been used in several studies focusing on health-related behaviour to predict both intention and actual behaviour (31–36). The interview guide focused on behavioural beliefs (beliefs about advantages and disadvantages of taking PrEP), normative beliefs (beliefs about how other people expect pregnant and breastfeeding women to behave with respect to whether or not to take PrEP), and control beliefs (how much control pregnant and breastfeeding women have over taking PrEP) (28). Women in our study were asked what they believed were the advantages and disadvantages of taking PrEP during pregnancy and breastfeeding; who they believed would approve or disapprove of their decision to take PrEP; and what they believed were potential facilitators and barriers to PrEP use. The interview guide also included questions aimed at exploring preferences for PrEP delivery in the target population.

The interview guide was piloted to ensure that questions were appropriately phrased and understood. Minor revisions were made based on this feedback and the revised guide was translated into two local languages (Nyanja and Bemba). SSIs were conducted by trained research assistants fluent in English, Nyanja, and Bemba. Prior to enrollment, interviewers described the study and emphasized the voluntary nature of participation. Written informed consent was obtained prior to any study activities. At the beginning of the interview, study staff described PrEP as medicine that HIV-negative people who feel that they might be at risk of acquiring HIV could take to prevent new infections. The following description was read to participants: “PrEP is the use of anti-retroviral drugs by HIV-uninfected people to protect them from getting infected with HIV. Daily oral PrEP is effective in preventing HIV infection when taken consistently.” Interviews were conducted in English and local languages (Nyanja, and Bemba), based on the participant's preference. All interviews were audio-recorded, and later transcribed and translated to English.

### Data analysis

Thematic analysis (37, 38), was used to identify beliefs and preferences that may influence PrEP uptake in antenatal and postnatal settings. Two study team members developed independent codebooks; differences were resolved through consensus and consolidated into a final version. Prior to data coding, we engaged in an iterative process of reading transcripts, which was accompanied by memoing, to identify common and unique content from the transcripts. This process was followed by categorizing content from each transcript under the sub-themes that were identified and later the broad themes (Theory of Planned Behavior constructs) based on the final codebook that was developed. Codes relating to advantages and disadvantages of using PrEP were categorized under attitude towards PrEP while codes relating to people who would approve or disapprove of the women's decision to use PrEP were categorized under subjective norm. Control beliefs comprised codes relating to presence of factors that would make it easy or difficult for women to use PrEP during pregnancy or breastfeeding. We also had several codes representing preferences for PrEP delivery during pregnancy and breastfeeding. Data were summarized using a framework matrix. The main themes and sub-themes are presented in Table 1. We used NVIVO v.12 (QSR International, Burlington, MA, USA) for data management and analysis.

**Table 1:** Respondent characteristics: HIV-negative pregnant and breastfeeding women (*N* = 24).

Characteristic	*n* (%)
Age in years, median (IQR)	24 (22–30)
Length of interview in minutes, median (range)	14 (8–43)
Marital status	
Never been married before	5 (21)
Married (living with partner)	18 (75)
Married (not living with partner)	1 (4)
Educational attainment	
Primary	7 (29)
Secondary	17 (71)
Employment	
Not working	17 (71)
Working for wages	1 (4)
Self employed	6 (25)
Maternal status	
Pregnant	13 (54)
Breastfeeding	11 (46)
Condom use in the last 30 days	
Never	18 (75)
Sometimes	6 (25)
Transactional sex in the last 30 days	
Yes	2 (8)
No	22 (92)
Partner HIV status	
Known	21 (88)
Unknown	3 (12)
Perceived HIV risk	
No risk	3 (13)
Low risk	13 (54)
Moderate risk	6 (25)
High risk	2 (8)
PrEP awareness before interview	
Yes	10 (42)
No	14 (58)

### Ethical approval

The study received approval from the University of Zambia Biomedical Research Ethics Committee (Lusaka, Zambia) and the Human Research Ethics Committee at the University of the Witwatersrand (Johannesburg, South Africa). Additional approvals were obtained from the Zambia National Health Research Authority and the Lusaka District Medical Office prior to study activation.

## Results

We conducted in-depth interviews with 24 HIV-negative pregnant and breastfeeding women. Baseline characteristics of study participants are presented in Table 1. Fifty percent of the women were aged below 24 years (IQR: 22–30 years) and the majority were married and living with their partners (18 of 24). Few women perceived themselves to be at high risk for HIV infection, the majority knew their partner's HIV status.

### Beliefs about PrEP

Most women (14 of 24) did not know about PrEP prior to the interview. Among the few that reported having knowledge about PrEP, some mistook PrEP for post-exposure prophylaxis while others described PrEP as treatment for sexually transmitted diseases. Beliefs that women held about PrEP were categorized into three broad themes based on the Theory of Planned Behaviour framework: (1) behavioural beliefs; (2) normative beliefs, and (3) control beliefs. We also asked specific questions about service delivery preferences for PrEP. Because these are related to control beliefs, these perspectives were included in that latter section. Table 2 is a summary of the broad and sub-themes from the interviews.

**Table 2:** Main themes and sub-themes from the study.

Behavioral beliefs	**Advantages of taking PrEP** • Protects pregnant/breastfeeding woman from contracting HIV • Protects the baby from contracting HIV **Disadvantages of taking PrEP** • Concerns about side effects to the woman • Concerns about side effects to the baby • Pill burden • Stigma/labelling from family, friends and community members	*“I would want to protect myself, and my baby as well, so that we do not contract HIV…. as you know…you can't just be trusting him just because you live with him… it's important to just drink the medicine so that you protect yourself.” (Participant 007).* *“I think I can be scared of taking this medication in the sense that I can be taking it [PrEP] without being sick and yet experiencing side effects like weight gain and so on. This can discourage me from taking this medication…” (Married, pregnant).*
Normative beliefs	**People who would approve or disapprove** • Partner • Other family members **What other people would think** • Stigma associated with HIV status • Promiscuity	*“My grandmother can disapprove, even my parents, like my mother… she would say no, you should just be using protection [condoms], things like that (Single, pregnant).* *“…my husband can disapprove. He can think that I can be sleeping around since I know that I won’t get sick because of taking PrEP. This would make it difficult for me to take PrEP.” (Married, breastfeeding).*
Control beliefs and preferences for PrEP service delivery	**Facilitators and Barriers to PrEP use** • Support from family and the community • Community awareness of PrE **Preferences for PrEP delivery** • Attitude of health care providers • Venue for collecting PrEP • Distance to the facility • Waiting time in the queue • Gender of health worker giving PrEP	*“There is too much stigma! If I tell my friend that I am taking this medication, she will go round telling people that I am sick [HIV positive]. This is what happens in our communities…the community does not support in any way.” (Married, breastfeeding).* “*Their attitude should be good; it just has to be good! Otherwise I would stop coming to collect PrEP if I found rude health care providers.” (Married, pregnant).* “*Any [whether male or female] is okay with me, as long as they have a good attitude.” (Participant 007).*

#### Behavioural beliefs about PrEP

Participants were asked what they thought were the advantages and disadvantages of taking oral PrEP every day during pregnancy and breastfeeding. The women expressed both positive and negative views. After learning about PrEP from the interviewer, participants felt that PrEP was good for them and their babies as it had the potential to protect them from acquiring HIV.

“*What I feel is the advantage of taking PrEP is that, PrEP protects us. It is good in the sense that one cannot easily be infected with HIV/AIDS. For example, my husband can have sex with another woman who may be HIV positive but when he sleeps with me, I may not be infected because PrEP will protect me from getting sick. So I feel oral PREP is good.” (Married, breastfeeding)*.

In addition, lack of trust for the partner was seen as a motivating factor to initiate PrEP during pregnancy and breastfeeding as a way of ensuring that they remained HIV-negative. According to some participants, trusting anyone, including one's own husband was a difficult thing to do. They argued that it was impossible for a woman to know all the whereabouts of her partner, adding that men would engage in multiple sexual relationships and in some instances, they would not even disclose to their spouses that they are on antiretroviral therapy. Women were of the view that, in such circumstances, PrEP could protect them from HIV infection if they had an unfaithful partner or had sex with someone who was HIV positive.

*“… it is so hard to trust someone these days. It is also hard to trust your own husband. This world is cruel, you can find that your husband is on ARVs [HIV treatment] and you do not know about it. So it is better for me to be taking PrEP in order to protect myself from contracting HIV/AIDS… taking PrEP can protect me from contracting HIV/AIDS.” (Married, pregnant)*.

Despite the positive impressions about PrEP in general, some women raised concerns about its use during pregnancy or breastfeeding. Fear of side effects, pill burden, and forgetfulness were some of the major issues that were seen as disadvantages of taking PrEP during pregnancy and breastfeeding. Women reported that they were given several other medications during pregnancy (e.g., iron supplements) and that adding more medications could be burdensome. They also reported that they did not want to deal with the side effects of PrEP.

*“I think I can be scared of taking this medication in the sense that I can be taking it [PrEP] without being sick and yet experiencing side effects like weight gain and so on. This can discourage me from taking this medication because I wouldn’t want to experience side effects when I know that I am not sick.” (Married, pregnant)*.

*“I think I have some reservations because I fear that my baby can be born prematurely because of taking PrEP.” (Single, pregnant)*

Other women did not view PrEP negatively in itself. The disadvantage they reported was linked to how others might view women who take PrEP when they are not sick [HIV-negative]

*I have not seen any disadvantage of taking PrEP. The only problem is with people who may end up laughing at you that you are taking medication yet you are not sick. This can discourage someone from taking PrEP (Participant 02)*.

#### Normative beliefs about PrEP use

We asked women to tell us about who they thought would approve and/or disapprove of their decision to take PrEP during pregnancy. Community and family members, especially male partners, were often cited. Stigma and being labeled to be on ART seemed to be a major concern that would hinder uptake of PrEP by pregnant and breastfeeding women.

*“There is too much stigmatization in the community. If I tell my friend that I am taking this medication, she will go round telling people that I am sick [HIV positive] and that is why am taking the medication when in the actual sense, I am protecting myself. This is what happens in our communities, we are used. The community does not support in any way.” (Married, breastfeeding)*.

Women also felt that people in their communities did not know much about PrEP, and that most community members would mistake PrEP for HIV treatment. The alleged lack of knowledge and stigma were seen as factors that would lead to pregnant and breastfeeding women being labeled as being HIV-positive once seen taking PrEP.

*“Sometimes you can decide to share with your friends about the medicine you are drinking [PrEP], they can think that you are taking ARVs [HIV treatment] and not PrEP, as you know knowledge levels are different among people in the community. There are people who are educated and those that are not educated and wouldn’t understand how PrEP works. They would say that PrEP is just the same as ARVs [HIV treatment] and that the difference is just the colour [of the pills].” (Married, pregnant)*.

Other participants viewed PrEP as something that would promote promiscuity among women. They argued that the mere knowledge that PrEP would protect one from contracting HIV would make women engage in risky behaviour including having multiple sexual partners.

*“I think people can take advantage of the fact that PrEP protects them from contracting HIV/AIDS. They can start misbehaving because they know that they will not get sick. This is the disadvantage I can think of.” (Married, breastfeeding)*.

#### Control beliefs about PrEP use

Pregnant and breastfeeding women were asked to describe circumstances that would make it easy and those that would make it difficult for them to take PrEP. For most women, whether or not to take PrEP would depend on approval from family members, including their male partners. In most cases, approval and support from their partners was seen as an important consideration when deciding to take PrEP. Women viewed the lack of support or approval from family members as a possible barrier to taking PrEP during pregnancy and breastfeeding.

*“I think my husband can make it difficult for me to take this PrEP. It can be difficult in that I do not take any pills. He may ask why I am taking PrEP because he knows I do not take any pills and he also knows that I use the injection as my contraceptive…he would wonder why I am taking this medication. He may also seek advice from his family and friends who could end up misleading him by making him believe that I am taking ARVs [HIV treatment]and not PrEP.” (Married, breastfeeding)*.

Some of the women stated that they would not take PrEP if their partners did not first approve.

*“…my husband can disapprove. He can think that I can be sleeping around since I know that I won”t get sick because of taking PrEP. I will need to explain to him the benefits of taking PrEP and if he is to understand then I can go ahead…but I would not drink it if he were to disapprove.” (Married, pregnant)*.

Although all acknowledged the important role of the partner, some women felt that they would still go ahead and take PrEP even without the support of their spouses. The final decision as to whether or not to take PrEP during pregnancy and breastfeeding was entirely up to them.

*“I think my husband can disapprove…. I can listen to him but that does not mean that I have to follow everything that he tells me to do. If at all I have decided to take the medication on my own, then he will have no right to stop me from taking it.” (Married, pregnant)*.

Daily dosing was also reported as a potential barrier to PrEP uptake. Women likened the idea of taking PrEP to taking oral contraceptives. They argued that there was no guarantee that they would remember to take PrEP on a daily basis when they were already having challenges taking oral contraceptives consistently during non-pregnant intervals. Some women were of the view that perhaps taking PrEP in form of injections would make it easier for them to use PrEP.

*“I think the issue of taking it [PrEP] daily is a problem because I can forget. It would be better if at all the medication was in form of an injection for 3 or 5 months. Taking tablets on a daily basis is dangerous because a person can forget to take it sometimes. We sometimes forget to take family planning pills, what would make us not to forget to take PrEP?” (Married, breastfeeding)*.

A few women made reference to structural factors when asked about barriers to PrEP use. Despite reporting that long distances and lack of transport money to the facility would make it difficult for them to take PrEP, most of the women felt that this would not stop them from using PrEP.

*“The distance does not matter as long as you know the importance of taking this medication. I can give an example of the people who are on ARVs [HIV treatment], they move for long distances in order for them to access ARVs. They even keep transport money or look for transport money in order for them to collect their medication. (Married, pregnant)*.

#### Preferences for PrEP delivery

Women were asked about health systems-related factors that could make it easy or difficult for them to use PrEP during pregnancy and breastfeeding. They described the attitude of health care providers as having the potential to either promote or hinder uptake of PrEP during pregnancy and breastfeeding. Women felt that health care providers needed to be polite, kind, patient, good-hearted and maintain confidentiality. They reported that a good attitude would encourage women to use PrEP. A bad attitude, on the other hand, could lead to discouragement for starting and maintaining PrEP.

*“Some nurses are rude and when they are talking, you are able to tell that they are rude. They should be kind to us because sometimes, you can get upset and some of us are emotional. They should be good and polite to us, because if they continue being rude, people can be discouraged from taking PrEP because it is like we are forcing them to do what they do not want to do.” (Married, breastfeeding)*.

Women were asked whether the gender of the health care provider dispensing PrEP mattered when it came to influencing uptake of PrEP during pregnancy and breastfeeding. For most women, gender did not seem to be an issue with respect to PrEP uptake. However, among those who had a preference for a specific gender, choices were based on different factors, including attitude and ability to relate to their experiences; some felt that female health care providers were more understanding compared to males.

*“They should be female because I am not comfortable with male health care providers…, I would feel shy when talking to a male health care provider. I can easily answer any question that a female nurse will ask me, but for a male nurse, I may not be free to answer accordingly.” (Married, breastfeeding)*.

We asked women whether waiting time at the health facility would be a source of concern when it came to using PrEP during pregnancy and breastfeeding. The majority of them reported that they would not want to be in the queue for a long time. The amount of time that women would be willing to wait at the facility in order to get PrEP ranged from 5 min to about 4 h. Most viewed waiting time as being dependent on how early one arrived at the facility and how many people they found already waiting in the queue. They also reported that waiting at the facility was something that they were used to doing and that it was normal practice to do so.

*“Queues will always be there; it all depends on how fast the queue is moving. If one does not want to spend too much time in the queue, then they have to come [to the facility] early.” (Married, pregnant)*.

Women preferred a health facility close to where they lived as this would make it easier for them to get to the facility to access PrEP. The amount of time they were willing to spend en route to the facility dispensing PrEP ranged from 15 min to 2 h. Although women preferred a facility that was closer to their homes, they reported that even if the facility was far, they would still make an effort to overcome this barrier in order to get PrEP. All women reported that they wanted to get PrEP from a health facility, either a clinic or hospital. When asked whether they would want PrEP to be delivered to their homes, women had differing views. Home delivery of PrEP would address the challenges associated with transportation to and from the health facility. It would also provide a positive opportunity for other community members to learn about PrEP.

*“I think it would be good if they delivered the medicine at my home. Home is actually better because other people can also get to learn about PrEP.” (Married, breastfeeding)*.

For other women, getting PrEP from the clinic was viewed negatively, as it would lead to discrimination and stigma. They expressed concern that queueing up for PrEP at the facility would make other people think that the women were actually on ART. For some women, getting PrEP from other facilities where people did not know them was seen as a better option.

*“If we collect from the clinic, isn”t it that we are supposed to be in a queue and other people who see us may think that we are sick or that we are getting ARVs [ART]? It is even better to collect from a clinic where people do not know you. People have a tendency of concluding.” (Married, breastfeeding)*.

### Summary of findings

**Figure 1:**
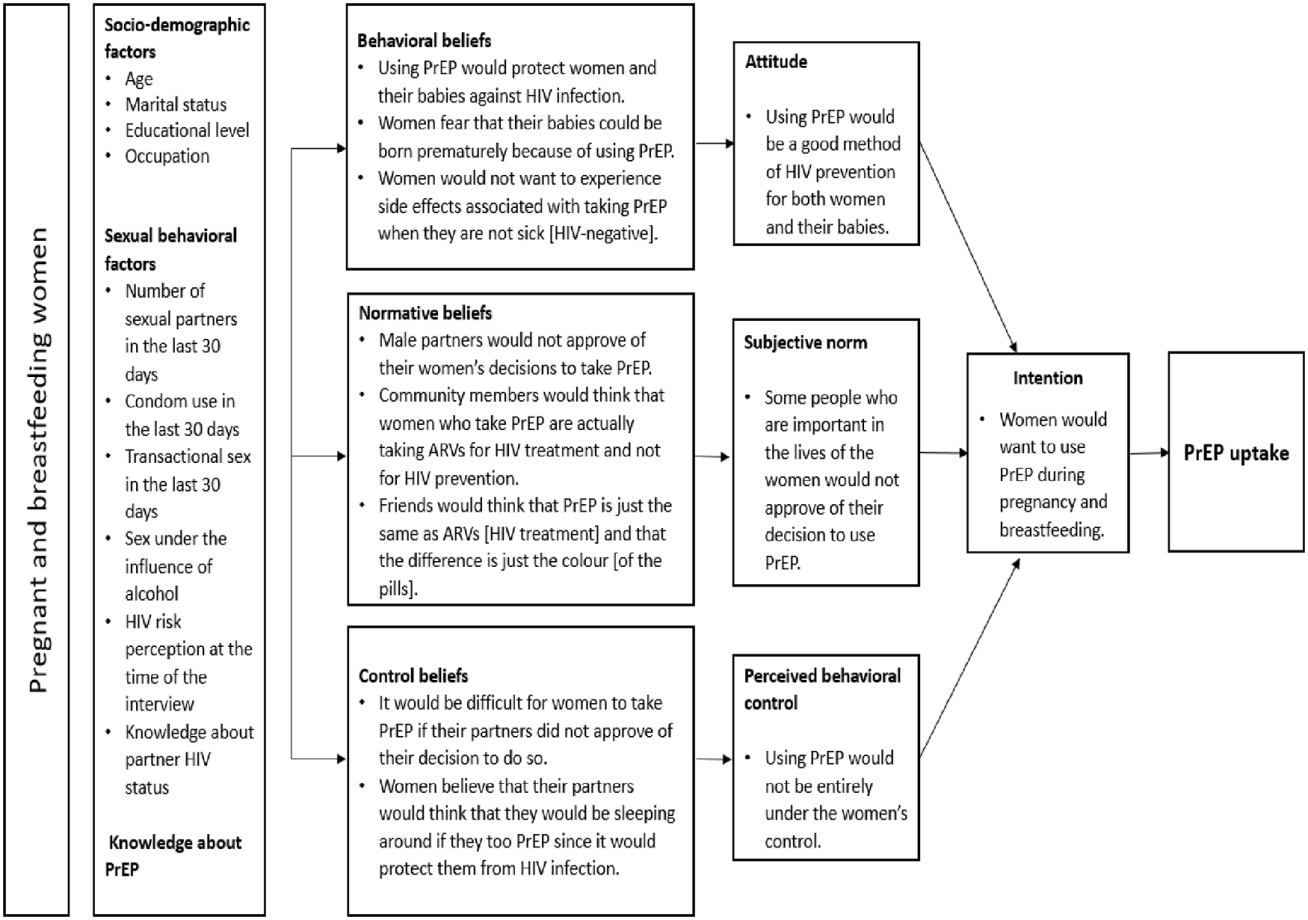
Mapping of findings onto the theory of planned behaviour conceptual framework.

In summary, we find that HIV-negative pregnant and breastfeeding women have positive attitudes towards PrEP use as a way of protecting themselves and their babies from HIV infection. Partners were cited as key referents when it comes to decision making on health-related matters and that their approval was critical if women were to use PrEP during pregnancy and breastfeeding. Women also believed that location for PrEP pick-up, attitude of health care providers, waiting time and distance to the facility may influence their decision to take PrEP during pregnancy and breastfeeding. These findings are mapped to the Theory of Planned Behavior conceptual model in Figure 1.

## Discussion

Women's perceptions about PrEP, particularly as they relate to the health of their children, have the potential to promote or hinder uptake during pregnancy and breastfeeding (39). Several of our key findings, particularly on women's behavioural beliefs and knowledge about PrEP echo results from other qualitative studies (7, 40), including one conducted by team members in Zambia and Malawi in 2017–2018 (41). Interestingly, despite the time that elapsed, many of the concerns remain the same, suggesting that more work is needed in outreach and education among prospective PrEP users.

Although women generally viewed PrEP as a good method to protect themselves and their infants from HIV infection, some participants expressed concerns that the availability of PrEP would encourage other women to engage in risky sexual behaviour. Health care providers in Kenya reported that PrEP users were sometimes confused, even frustrated, with their insistence on using condoms in addition to PrEP (40). Similar fears around the potential for PrEP to reduce condom use were reported in Eswatini (42). Davey et al. also noted that the perceptions that PrEP would lead to more risk or more condom-less sex was a potential barrier to uptake and adherence (39).

Stigma and misinformation are often cited as barriers to PrEP use (43). From our results, women feared that, if they took PrEP, people would label them as being HIV-positive and not being truthful about their HIV status. They attributed this to inadequate knowledge about PrEP in their communities. In a similar study conducted in Uganda, South Africa and Zimbabwe, participants mentioned that they would refrain from taking PrEP because of its association with antiretroviral drugs and HIV-related stigma. This was a key barrier to uptake as participants linked taking daily tablets to people living with HIV (44). HIV-related stigma is common in Zambia (45, 46) and could negatively impact PrEP uptake, adherence, and retention in care at a time when women need it the most to ensure that their infants and themselves stay HIV-negative. Deliberate efforts aimed at developing community-based education programs with a focus on demystifying PrEP may have a significant impact on PrEP uptake among HIV-negative pregnant and breastfeeding women.

The level of male partner support may influence women's decision to take PrEP during pregnancy and breastfeeding. Evidence suggests that men are generally viewed as head of house and ultimate decision makers who are actively involved in health-related decision-making during pregnancy and breastfeeding (47, 48). Our study found that women had divergent views about who would approve of their decision to take PrEP during pregnancy and breastfeeding. The key referents, however, were mostly women's partners. Similar findings have been reported in similar settings. In a study that contextualized male roles and participation in PMTCT programs in Malawi and Zambia, for instance, both men and women reported that they had to consult and seek approval from their partners on decisions that related to their health (47). Other studies also reported that men were the primary advisors and key decision-makers on health-related decisions during pregnancy and breastfeeding (41, 49, 50).

From our findings, male partners may, to a greater extent, determine actual uptake of PrEP by women during pregnancy and breastfeeding. Similar findings were reported elsewhere (50). Such gender-based power differentials, specifically the lack of autonomy among women to make decisions concerning their health may present a barrier to PrEP uptake during pregnancy and breastfeeding. Male involvement in promoting PrEP uptake among pregnant and breastfeeding women could increase the use of PrEP in this target population. In some circumstances, such engagement may be challenging and this should be recognized. Programs should offer these women additional support for HIV prevention, whether through PrEP or other proven modalities.

Participants in our study expressed concern about the gender of health care providers. Interestingly, however, gender was rarely discussed in isolation, but rather with reference to the attitude of health care providers. Women who preferred to be attended to by male health care providers viewed female health care providers negatively. This finding is supported by prior research where respondents complained about the poor attitude of health care providers, especially female nurses being disrespectful, rude and using abusive words (48). Participants in our study reported that health care providers who had a bad attitude would make it difficult for women to use PrEP during pregnancy and breastfeeding. Some women felt that they would not use PrEP if the health care providers dispensing PrEP were rude to them. Our findings on the impact of health care provider attitude on PrEP uptake are consistent with other studies (44).

Distance and waiting time are structural factors that may have a negative impact on PrEP uptake and retention in care. Women in our study reported that having a health facility nearer to where they lived would make it easier for them to take PrEP. A study in Uganda also found that walking time to the clinic of thirty minutes or greater was associated with decreased odds of uptake of PrEP (51). Although distance was cited as a barrier in our current study, it is possible that its effect on PrEP uptake could be indirect as women may have taken into consideration the transport-related costs of remaining in PrEP care if they decided to initiate PrEP in the future. Waiting time in the queue was also cited as a potential barrier to PrEP uptake. Women did not want to wait for a long time at the facility in order to collect PrEP. Similar to our findings, a study conducted in Botswana, Tanzania and Uganda reported that lost wages due to waiting time was a barrier to adherence among individuals on antiretroviral therapy (52). These findings are corroborated by those from a recent study conducted by our team (36).

### Strengths and limitations

The main strength of this study is our use of an established theoretical framework to determine potential barriers and facilitators to PrEP use in pregnant and breastfeeding women. The perspectives shared are specific to this population, providing important insights during an important period in women's lives. At the same time, we also recognize some limitations. First, this study focused on individual-level cognitions and did not explore other unconscious influences that could potentially account for variances in PrEP uptake behavior. However, evidence suggests that interventions resulting in large changes in intention are likely to also change behavior (53). Second, our results are a mix of beliefs held by women who were and those who were not knowledgeable about PrEP prior to study participation. Responses of the latter could be prone to social desirability bias. Third, we enrolled only pregnant and breastfeeding women in our study. While this was viewed as a strength overall, we acknowledge our limited ability to compare these responses to those of other women outside of this window. Further, it is possible that some participants' responses may not have specifically focused on PrEP use during pregnancy and breastfeeding intervals, owing to their limited awareness about PrEP prior to the interviews and lack of experience using PrEP. Nevertheless, their perspectives still offer useful insights on beliefs likely to influence PrEP uptake in antenatal and postnatal settings. Exploring and documenting the lived experiences of women who have used PrEP during pregnancy and breastfeeding could enhance our understanding of facilitators and barriers to PrEP uptake and onward adherence and retention in care in this population. This represents a gap for further research. Fourth, the study was based at a single facility and, therefore, our results may not be generalized to the larger antenatal and postnatal populations in Zambia—or in the sub-Saharan African region. As with all qualitative studies, we instead focus on the depth of participant beliefs and preferences to inform future PrEP implementation in antenatal settings. Fifth, PrEP was not readily accessible at the study site at the time this study was implemented. The hypothetical nature of PrEP in the study only provided partial understanding of facilitators and barriers to PrEP uptake as the views of women interviewed may differ from those of women who have experience taking PrEP during pregnancy and breastfeeding. Nevertheless, the positive attitude towards PrEP use among participants was reassuring and provides an avenue to promote uptake of PrEP during pregnancy and breastfeeding.

## Conclusion

Our study suggests that HIV-negative pregnant and breastfeeding women have positive attitudes towards PrEP but barriers to PrEP uptake are multifaceted. To ensure that PrEP implementation in antenatal settings is successful, there is need to address the inadequate knowledge about PrEP among pregnant and breastfeeding women—and the broader community as well. Interventions that promote male involvement in female-initiated methods for HIV prevention may result in improved knowledge and a more supportive attitude among men towards women who wish to use PrEP during pregnancy and breastfeeding. Addressing contextual barriers—including distance, waiting time at the facility, and health care provider attitude—could have a significant impact on PrEP uptake. Above all, exploring the lived experiences of pregnant and breastfeeding women who have used PrEP before would be critical to the design of effective PrEP implementation strategies in this population in need.

## Data Availability

The datasets presented in this article are not readily available because the qualitative nature of these data make it difficult for us to share them publicly, mainly owing to the potential for identification. However data will be made available through the University of Zambia, School of Public Health (deansoph@unza.zm) for researchers who meet the criteria for access to confidential data. Requests to access the datasets should be directed to Dean-School of Public Health, deansoph@unza.zm.
